# Supplementary material for: Cardiac Involvement in Patients with Multisystem Inflammatory Syndrome in Children (MIS-C) in Poland
Source: Biomedicines. 2023 Apr 23;11(5):1251. doi: 10.3390/biomedicines11051251 (PMC10215748; doi:10.3390/biomedicines11051251)
Supplement: Supplementary file 1 [file biomedicines-11-01251-s001.zip › biomedicines-2307055-supplementary.pdf]

# Supplementary material for:

## Cardiac involvement in patients with multisystem inflammatory syndrome in children (MIS-C) in Poland

Kamila M. Ludwikowska[1], Nafeesa Moksud[2], Paweł Tracewski[3], Mateusz Sokolski[4*], Leszek Szenborn[1*]

1) Department of Pediatric Infectious Diseases, Wroclaw Medical University, Ludwika Pasteura 1, 50-367 Wrocław Poland
2) Laboratory of Genetics and Epigenetics of Human Diseases, Hirszfeld Institute of Immunology and Experimental Therapy, Polish Academy of Sciences, Rudolfa Weigla 12, 53-114, Wrocław, Poland
3) Department of Pediatric Cardiology, Regional Specialist Hospital in Wroclaw, Research and Development Center, Kamieńskiego 73a, 51-124 Wrocław, Poland
4) Institute of Heart Diseases, Wroclaw Medical University, Borowska 213, 50-556 Wroclaw, Poland

* last co-author

Table S1. The characteristics of the study group and multisystem inflammatory syndrome course by age group.

| | | Overall Cohort | <5 years | 5-11 years | 12-18 years | p-value |
|---|---|---|---|---|---|---|
| Counts (%) or Median (25th-75th percentile) | | 498 (100%) | 137 (28%) | 251 (50%) | 110 (22%) | |
| **Demographical features** | | | | | | |
| Male sex | | 314 (64%) | 76 (56%) | 159 (64%) | 79 (73%) | **0.02** |
| Age | | 8.25 (4.67-11.62) | 3.19 (1.93-4.18) | 8.60 (6.66-10.48) | 13.76 (12.95-15.17) | - |
| Race | White | 496 (99.6%) | 135 (99%) | 251 (100%) | 110 (100%) | 0.12 |
| | Asian | 2 (0.4%) | 2 (1%) | 0 (0%) | 0 (0%) | |
| Weight | kg | 28.0 (18.5-46.0) | 14.7 (12.2-18.0) | 30.0 (23.0-38.0) | 57.0 (49.0-69.0) | **0.00** |
| Height | cm | 132.(113-154) | 100 (90-108) | 134 (123-143) | 166 (160-174) | **0.00** |
| BMI | kg/m^2 | 16.6 (14.9-19.6) | 15.2 (14.4-16.5) | 16.1 (14.7-19.0) | 20.6 (18.3-23.1) | **0.00** |

| | | | | | | |
|---|---|---|---|---|---|---|
| BMI centiles | | 51.9 (21.1-80.7) | 40.9 (17.4-77.0) | 50.7 (20.6-82.6) | 65.9 (30.8-83.9) | **0.01** |
| BMI groups | Normal | 324 (73%) | 85 (71%) | 163 (73%) | 76 (75%) | 0.19 |
| | Obese | 33 (7%) | 7 (6%) | 18 (8.%) | 8 (8%) | |
| | Overweight | 60 (14%) | 13 (11%) | 32 (14%) | 15 (15%) | |
| | Underweight | 28 (6%) | 14 (12%) | 11 (5%) | 3 (3%) | |
| **Comorbidities*** | | | | | | |
| None | | 447 (93%) | 126 (95%) | 223 (93%) | 98 (91%) | 0.47 |
| **Signs and symptoms** | | | | | | |
| Days of fever | | 7 (6-9) | 7 (6-8) | 7 (6-9) | 7 (6-9) | 0.12 |
| Gastrointestinal symptoms | | 436 (89%) | 110 (83%) | 224 (90%) | 102 (94%) | **0.03** |
| Mucocutaneous involvement | | 475 (96%) | 130 (96%) | 243 (97%) | 102 (94%) | 0.42 |
| Upper respiratory symptoms | | 173 (37%) | 48 (39%) | 80 (33%) | 45 (42%) | 0.23 |
| Lower respiratory symptoms | | 217 (46%) | 46 (36%) | 105 (44%) | 66 (63%) | **0.00** |
| Neurological symptoms | | 383 (91%) | 111 (93%) | 188 (89%) | 86 (92%) | 0.41 |
| Osteoarticular and muscle involvement | | 172 (37%) | 32 (27%) | 94 (39%) | 46 (44%) | **0.02** |
| Systemic oedema | | 2 (2%) | 0 (0%) | 1 (2%) | 1 (4%) | 0.70 |
| **Vital signs at peak of disease** | | | | | | |
| AVPU other than A | | 61 (14%) | 18 (15%) | 28 (12%) | 15 (15%) | 0.71 |
| Heartrate (beats/minute) | Max | 135 (120-150) | 145 (130-160) | 130 (120-141) | 128 (110-140) | **0.00** |
| | Min | 69 (57-81) | 80 (68-96) | 68 (56-80) | 62 (50-70) | **0.00** |
| Prolonged CRT (>2s) | | 53 (14%) | 12 (12%) | 25 (14%) | 16 (20%) | 0.30 |
| Systolic blood pressure (mmHg) | | 85 (76-93) | 85 (75-93) | 84 (78-92) | 84 (75-95) | 0.59 |
| Hypotension | | 206 (49%) | 27 (25%) | 118 (56%) | 61 (62%) | **0.00** |
| Max. respiratory rate (breaths/min) | | 25 (20-30) | 28 (22-37) | 25 (20-30) | 25 (20-30) | 0.08 |
| Min. SatO2 (%) | | 96 (93-97) | 96 (94-98) | 96 (93-98) | 95 (92-97) | **0.02** |
| **Laboratory test results at the peak of disease** | | | | | | |
| WBC max (10^3/µl) | | 15.4 (11.1-20.6) | 16.8 (12.3-21.8) | 14.9 (10.9-20.3) | 14.7 (11.5-20.0) | 0.09 |
| Neutrophils max (10^3/µl) | | 10.1 (7.3-14.9) | 9.6 (6.4-14.6) | 9.9 (7.3-14.5) | 12.3 (8.8-16.2) | **0.05** |
| Lymphocytes min (10^3/µl) | | 1.0 (0.6-1.7) | 1.7 (1.0-3.0) | 0.9 (0.6-1.4) | 0.6 (0.5-1.0) | **0.00** |
| Hb min (g/dl) | | 10.4 (9.5-11.2) | 9.7 (9.0-10.4) | 10.5 (9.7-11.2) | 10.9 (10.1-11.9) | **0.00** |
| Hct min (%) | | 30.0 (27.6-32.3) | 28.6 (26.6-30.5) | 30.3 (27.9-32.5) | 31.7 (29.5-34.0) | **0.00** |
| PLT min (10^3/µl) | | 163 (109-228) | 174 (111-265) | 164 (110-224) | 148 (107-203) | 0.09 |
| CRP max (mg/l) | | 169 (98-242) | 139 (88-208) | 169 (100-242) | 209 (121-289) | **0.00** |
| ESR max (mm/h) | | 57.0 (35.0-77.0) | 60.0 (36.0-72.0) | 58.0 (35.0-81.0) | 50.0 (33.0-77.0) | 0.73 |
| Serum Glucose min (mg/dl) | | 86 (77-98) | 85 (76-98) | 85 (77-96) | 88 (79-103) | 0.10 |
| Serum Glucose max (mg/dl) | | 112 (97-135) | 107 (93-127) | 113 (96-136) | 118 (102-137) | 0.05 |
| Fibrinogen max (g/l) | | 5.7 (4.6-7) | 5.5 (4.5-6.7) | 5.6 (4.5-6.9) | 6.3 (5.3-7.3) | **0.03** |
| LDH max (U/l) | | 295 (242-344) | 309 (264-363) | 289 (239-330) | 272 (234-341) | **0.02** |
| Procalcitonin max (ng/ml) | | 4.1 (1.4-12.8) | 4.9 (2.2-10.4) | 3.7 (1.3-12.3) | 3.4 (1.1-13.4) | 0.61 |
| Ferritin max (ug/l) | | 387 (216-645) | 270 (156-421) | 413 (249-644) | 511 (238-929) | **0.00** |
| Albumins min (g/dl) | | 2.9 (2.6-3.4) | 2.9 (2.6-3.4) | 2.9 (2.5-3.4) | 3.0 (2.6-3.5) | 0.29 |
| Sodium min (mmol/l) | | 133 (130-135) | 134 (132-136) | 133 (130-135) | 133 (129-135) | **0.03** |
| D-dimer max (mg/l) | | 3.7 (2.0-5.6) | 3.8 (1.7-5.3) | 3.7 (2.1-6.1) | 3.5 (2.2-5.4) | 0.51 |
| IL-6 max (pg/ml) | | 122 (50-524) | 122 (66-514) | 128 (26-549) | 133 (38-486) | 0.91 |
| eGFR min (ml/min/1.73m^2) | | 105 (85-126) | 112 (90-137) | 107 (88-125) | 88 (65-118) | **0.00** |
| BNP max (pg/ml) | | 1070 (264-5245) | 329 (87-1458) | 1577 (342-5580) | 2551 (448-11480) | **0.00** |
| NT-proBNP max (pg/ml | | 4744 (1462-11479) | 4737 (1166-15005) | 4136 (1592-9679) | 6340 (2718-16000) | 0.14 |
| Troponin elevated | | 97 (50%) | 5 (16%) | 53 (51%) | 39 (64%) | **0.00** |
| **Echocardiography features** | | | | | | |
| Any CAA | | 36 (10.5%) | 17 (17.5%) | 12 (7.4%) | 7 (8.4%) | **0.04** |
| Any Contractility | | 155 (41.4%) | 27 (28.1%) | 75 (39.9%) | 53 (58.9%) | **0.00** |

| | | | | | |
|---|---|---|---|---|---|
| Any Pericardial Effusion | 47 (13.4%) | 13 (13.4%) | 23 (13.5%) | 11 (13.1%) | 1.00 |
| Any Valvular Insufficiency | 190 (48.2%) | 45 (42.5%) | 104 (53.1%) | 41 (44.6%) | 0.15 |
| Decreased LVEF (%) | 132 (35.6%) | 22 (22.7%) | 65 (38.9%) | 45 (50.6%) | **0.00** |
| LVEF in Initial Echo (%) | 55.00 (46.00-64.00) | 55.00 (46.00-64.00) | 54.00 (46.00-63.20) | 55.00 (46.00-65.00) | 0.38 |
| LVEF in Follow-up Echo (%) | 55.80 (49.00-66.00) | 55.00 (45.00-66.00) | 58.00 (49.00-67.00) | 51.00 (48.00-58.00) | 0.12 |
| **Management** | | | | | |
| PICU treatment | 32 (6.6%) | 3 (2.3%) | 17 (7.0%) | 12 (11.1%) | **0.02** |
| IVIG administered | 447 (91.0%) | 128 (94.1%) | 223 (89.6%) | 96 (90.6%) | 0.31 |
| GCS administered | 344 (71.5%) | 86 (63.7%) | 170 (71.7%) | 88 (80.7%) | **0.01** |
| ASA administered | 438 (100.0%) | 125 (100.0%) | 224 (100.0%) | 89 (100.0%) | **-** |
| Heparin administered | 128 (37.5%) | 28 (30.1%) | 61 (37.0%) | 39 (47.0%) | 0.07 |

Abbreviations: AVPU, alert, verbal, pain, unresponsive; ASA, acetylsalicylic acid; BMI, body mass index; BNP, brain natriuretic peptide; CRP, C reactive protein; CRT, capillary refill time; eGFR, estimated glomerular filtration rate; ESR, erythrocyte sedimentation rate; GCS, glucocorticosteroids; Hb, haemoglobin; Hct, haematocrit; Il-6, interleukin 6; IQR, interquartile range; IVIG, intravenous immunoglobulins; LDH, lactate dehydrogenase; LVEF, left ventricular ejection fraction; min, minutes; n, number; NT-proBNP, N-terminal prohormone for brain natriuretic peptide; PICU, paediatric intensive care unit; PLT, platelet count; s, seconds; SatO2, oxygen saturation; WBC, white blood count

*other than cardiovascular

Gastrointestinal symptoms encompassed: nausea, vomiting, diarrhoea or abdominal pain; mucocutaneous involvement encompassed: rash, erythema at BCG site, conjunctivitis, hands and feet erythema or oedema, digital peeling, inflammation of the oral cavity or cervical lymphadenopathy; upper respiratory symptoms encompassed: coryza or sore throat; lower respiratory symptoms encompassed: cough, breathing effort, chest pain and swallowing difficulty; neurological involvement encompassed: meningeal signs, lethargy, seizures, headache, muscle hypotension, peripheral nerve paralysis, paresis, loss of smell or taste, photophobia, agitation or skin hyperesthesia; osteoarticular and muscle involvement encompassed: arthritis, arthralgia or muscle pain

Max stands for the maximal value of the result obtained

Min stands for the minimal value of the result obtained

## Table S2. Initial and follow-up echo findings in patients with multisystem inflammatory syndrome in children (MIS-C)

| **Feature**<br>Counts (%) or Median (25th-75th percentile) | | **Initial Echo**<br>n=498 | **Follow up Echo**<br>n=376 |
|---|---|---|---|
| **Echo findings** | | | |
| **Coronary artery abnormality** | | | |
| CAA | None | 317 (94%) | 317 (94%) |
| | Dilation | 5 (2%) | 6 (2%) |
| | Aneurysms | 14 (4%) | 13 (4%) |
| **Contractility** | | | |

| | | | |
|---|---|---|---|
| Contractility described as abnormal | | 100 (29%) | 66 (19%) |
| Heart apex rounding or segmental contraction disfunction | | 12 (4%) | 20 (6%) |
| Left Ventricular Function[#] | | | |
| LVEF (%) | First | 55 (46-64) | |
| | Follow-up | | 56 (49-66) |
| Decreased LVEF | | 91 (26%) | 50 (15%) |
| **Valves** | | | |
| Valve insufficiency described* | None | 237 (70%) | 256 (75%) |
| | Any | 104 (31%) | 85 (25%) |
| | IMV | 84 (54%) | 47 (55%) |
| | ITV | 6 (4%) | 6 (7%) |
| | IPV | 2 (1%) | 0 (0%) |
| | IAoV | 1 (1%) | 0 (0%) |
| | IMV+ITV | 55 (35%) | 25 (29%) |
| | IMV+IPV | 1 (1%) | 0 (0%) |
| | IMV+IAoV | 1 (1%) | 1 (1%) |
| | ITV+IPV | 2 (1%) | 0 (0%) |
| | IMV+ITV+IPV | 3 (2%) | 5 (6%) |
| | IMV+ITV+IPA | 2 (1%) | 0 (0%) |
| | IMV+ITV+IAoV | 0 (0%) | 1 (1%) |
| Level of valve insufficiency (1-3) | None | 235 (70%) | 253 (76%) |
| | 1 | 65 (20%) | 55 (17%) |
| | 2 | 29 (9%) | 21 (6%) |
| | 3 | 5 (2%) | 5 (2%) |
| **Pericardial effusion** | | | |
| Pericardial effusion | | 25 (7%) | 16 (5%) |

Abbreviations: CAA, coronary artery abnormality; Echo, echocardiography; EF, ejection fraction; IAoV, aortic valve insufficiency; IMV, mitral valve insufficiency; IPV, pulmonary valve insufficiency; ITV, tricuspid valve insufficiency; LVEF, left ventricular ejection fraction

#LVEF was evaluated using Simpson's biplane method

* Represents the number of defects counted

Table S3. Changes between the baseline and follow-up echocardiography findings in patients with multisystem inflammatory syndrome in children (MIS-C) and two echo descriptions available

| **Patients with both Initial and Follow-up Echo** (n=337) | | | |
|---|---|---|---|
| **Feature**<br>Counts (%) or Median (25th-75th percentile) | **Initial Echo** | **Follow-up Echo** | **p-value** |
| **Coronary artery abnormality** | | | |
| None | 315 (94.3%) | 315 (94.3%) | 1.00 |
| Dilation | 5 (1.5%) | 6 (1.8%) | |
| Aneurysms | 14 (4.2%) | 13 (3.9%) | |
| **Contractility** | | | |
| Contractility described as abnormal | 97 (29.0%) | 63 (18.8%) | **0.00** |

| | | | | |
|---|---|---|---|---|
| Heart apex rounding or segmental contraction disfunction | | 11 (3.3%) | 20 (5.9%) | 0.08 |
| LVEF# (%) | First Follow-up | 55.5 (47-64) | 56.5 (49.5-68.5) | **0.02** |
| Decreased LVEF | | 87 (25.8%) | 47 (13.9%) | **0.00** |
| **Valves** | | | | |
| Any valve insufficiency described | | 102 (30.4%) | 82 (24.4%) | **0.04** |
| Level of valve insufficiency (1-3) | None | 232 (70.3%) | 251 (76.1%) | - |
| | 1 | 65 (19.7%) | 55 (16.7%) | |
| | 2 | 28 (8.5%) | 19 (5.8%) | |
| | 3 | 5 (1.5%) | 5 (1.5%) | |
| **Pericardial effusion** | | | | |
| Pericardial effusion | | 26 (7.7%) | 15 (4.5%) | 0.09 |

Abbreviations: CAA, coronary artery abnormality; Echo, echocardiography; EF, ejection fraction; IAoV, aortic valve insufficiency; IMV, mitral valve insufficiency; IPV, pulmonary valve insufficiency; ITV, tricuspid valve insufficiency; LVEF, left ventricular ejection fraction

#LVEF was evaluated using Simpson's biplane method

Table S4. Comparison between patients with and without echocardiographic abnormalities in the course of the multisystem inflammatory syndrome in children (MIS-C).

| | | **Whole cohort** | **Patients with no abnormalities on first and follow-up echo** | **Patients with abnormalities on first or follow-up echo** | **p-value** |
|---|---|---|---|---|---|
| Feature<br>Counts (%) or Median (25th-75th percentile) | | n=498 | n=121 | n=264 | |
| **Demographical features** | | | | | |
| Male sex | Male | 314 (64%) | 69 (58%) | 175 (67%) | 0.09 |
| Age | | 8.3 (4.7-11.6) | 8.3 (4.4-11.1) | 8.7 (5.0-12.1) | 0.16 |
| Age Group | <5 | 137 (28%) | 38 (31%) | 64 (24%) | 0.27 |
| | 5-11 | 251 (50%) | 59 (49%) | 133 (50%) | |
| | 12-18 | 110 (22%) | 24 (20%) | 67.00 (25%) | |
| Weight (kg) | | 28.0 (18.5-46.0) | 28.0 (18.5-42.0) | 31.00 (19.00-50.00) | **0.05** |
| Height (cm) | | 132 (113-154) | 132.0 (112.0-150.0) | 134.00 (114.00-156.00) | 0.16 |
| BMI (kg/m^2) | | 16.6 (14.9-20.0) | 16.6 (14.8-19.4) | 16.83813 (1503.8%) | 0.10 |
| BMI groups | normal | 324 (73%) | 83 (77%) | 177 (74.7%) | 0.94 |
| | obese | 33 (7%) | 7 (7%) | 16 (6.8%) | |
| | overweight | 60 (14%) | 12 (11%) | 32 (13.5%) | |

| | | | | |
|---|---|---|---|---|
| underweight | 28 (6%) | 6 (6%) | 12 (5.1%) | |
| Comorbidities | 34 (7%) | 7 (6%) | 0.00 (0.00-0.00) | 0.53 |
| **Signs and symptoms** | | | | |
| Days of fever | 7 (6-9) | 7 (6-9) | 7 (6-8) | 0.43 |
| Gastrointestinal symptoms | 436 (89%) | 108 (91%) | 25 (10%) | 1.00 |
| Mucocutaneous involvement | 475 (96%) | 117 (98%) | 233 (90%) | 0.51 |
| Upper respiratory symptoms | 173 (37%) | 40 (36%) | 9 (3%) | 0.91 |
| Lower respiratory symptoms | 217 (46%) | 45 (42%) | 253 (97%) | 0.09 |
| Neurological symptoms | 383 (91%) | 91 (83%) | 166 (65%) | **0.00** |
| Osteoarticular and muscle involvement | 172 (37%) | 44 (38%) | 91 (35%) | 0.91 |
| Systemic oedema | 2 (2%) | 0 (0%) | 124 (48%) | 1.00 |
| Particular symptoms | | | | |
| Arthritis | 19 (4%) | 4 (3%) | 12 (4.7%) | 0.78 |
| Conjunctivitis | 379 (78%) | 97 (82%) | 204 (78.8%) | 0.49 |
| Eyelid swelling | 70 (31%) | 7 (32%) | 44 (32.4%) | 1.00 |
| Rhinitis | 60 (13%) | 10 (9%) | 29 (11.3%) | 0.47 |
| Oral inflammation | 329 (68%) | 81 (7%) | 180 (69.8%) | 0.90 |
| Breathing effort | 101 (21%) | 17 (15%) | 70 (27.0%) | **0.01** |
| Chest pain | 65 (14%) | 13 (12%) | 44 (17.3%) | 0.21 |
| Abdominal pain | 384 (80%) | 97 (84%) | 206 (81.1%) | 0.66 |
| Nausea | 283 (59%) | 73 (62%) | 151 (59.7%) | 0.65 |
| Diarrhoea | 270 (55%) | 74 (63%) | 147 (56.5%) | 0.31 |
| **Vital signs at admission** | | | | |
| AVPU other than A | 23 (5%) | 2 (2%) | 16 (7%) | 0.07 |
| Heartrate (beats/minute) | 120 (105-138) | 120 (100-140) | 124 (110-140) | 0.14 |
| Prolonged CRT (>2s) | 50 (13%) | 15 (16%) | 27 (13%) | 0.59 |
| Systolic blood pressure (mmHg) | 99 (89-109) | 101 (93-110) | 98 (87-108) | **0.03** |
| Hypotension | 64 (17%) | 9 (9%) | 46 (22.1%) | **0.01** |
| Respiratory rate (breaths/minute) | 20 (18-26) | 20 (18-26) | 22 (18-28) | **0.04** |
| SatO2 (%) | 98 (96-99) | 98 (96-99) | 98 (97-99) | 0.61 |
| **Laboratory test results at admission** | | | | |
| WBC (10^3/µl) | 9.6 (6.7-13.0) | 9.7 (6.5-12.7) | 9.71 (6.65-13.66) | 0.87 |
| Neutrophils (10^3/µl) | 7.6 (4.9-10.4) | 7.8 (5.0-10.0) | 7.9 (4.8-11.0) | 0.90 |
| Lymphocytes (10^3/µl) | 1.0 (0.7-1.8) | 1.2 (0.7-1.8) | 0.9 (0.6-1.5) | 0.15 |
| Hb (g/dl) | 11.7 (10.7-12.7) | 11.7 (10.6-12.6) | 11.7 (10.7-12.7) | 0.91 |
| Hct (%) | 34.0 (31.0-36.7) | 33.8 (31.0-37.0) | 33.8 (30.5-36.6) | 0.38 |
| PLT (10^3/µl) | 179 (130-248) | 190 (139-250) | 175 (122-238) | 0.21 |
| CRP (mg/l) | 144 (84-206) | 141 (84-188) | 150 (87-223) | 0.12 |
| ESR (mm/h) | 46 (30-66) | 47 (30-71) | 47 (30-67) | 0.93 |
| Fibrinogen (g/l) | 5.5 (4.5-6.6) | 5.3 (4.5-6.5) | 5.6 (4.6-6.6) | 0.73 |
| LDH (U/l) | 281 (235-334) | 266 (227-320) | 283 (242-336) | 0.28 |
| Procalcitonin (ng/ml) | 2.8 (1.1-7.6) | 1.8 (0.8-5.1) | 3.5 (1.3-12.8) | **0.00** |
| Ferritin (ug/l) | 331 (198-567) | 316 (177-532) | 401 (212-708) | 0.07 |

| | | | | |
|---|---|---|---|---|
| Serum Glucose (mg/dl) | 100 (89-117) | 99.4 (88-116) | 102 (89-117) | 0.94 |
| Albumins (g/dl) | 3.4 (3.0-3.8) | 3.4 (2.8-3.7) | 3.4 (2.9-3.7) | 0.88 |
| Sodium (mmol/l) | 134 (132-136) | 135 (132-137) | 133 (131-136) | **0.00** |
| D-dimer (mg/l) | 2.6 (1.5-4.5) | 2.4 (1.4-4.4) | 2.9 (1.7-4.8) | 0.08 |
| IL-6 (pg/ml) | 105 (45-321) | 105 (45-303) | 113 (30-351) | 0.89 |
| eGFR (ml/min/1.73m^2) | 109 (86-132) | 113 (95-135) | 104 (82-132) | 0.23 |
| BNP (pg/ml) | 287 (114-1006) | 449 (155-865) | 302 (94-1657) | 0.36 |
| NT-proBNP (pg/ml) | 2176 (486-7426) | 2010 (651-5372) | 3399 (641-10317) | 0.08 |
| Troponin elevated | 63 (26%) | 12 (16%) | 46 (35%) | **0.00** |
| **Management** | | | | |
| PICU treatment | 32 (7%) | 6 (5%) | 25 (10%) | 0.16 |
| IVIG administered | 447 (91%) | 111 (93%) | 253 (97%) | 0.12 |
| GCS administered | 344 (72%) | 82 (72%) | 202 (78%) | 0.19 |
| ASA administered | 438 (100%) | 110 (100%) | 236 (100%) | |
| Heparin administered | 128 (38%) | 40 (39%) | 76 (44%) | 0.45 |

Abbreviations: AVPU, alert, verbal, pain, unresponsive; ASA, acetylosalicylic acid; BMI, body mass index; BNP, brain natriuretic peptide; CRP, C reactive protein; CRT, capillary refill time; eGFR, estimated glomerular filtration rate; ESR, erythrocyte sedimentation rate; GCS, glucocorticosteroids; Hb, hemoglobin; Hct, hematocrit; Il-6, interleukin 6; IQR, interquartile range; IVIG, intravenous immunoglobulins; LDH, lactate dehydrogenase; min, minutes; n, number; NT-proBNP, N-terminal prohormone for brain natriuretic peptide; PICU, pediatric intensive care unit; PLT, platelet count; s, seconds; SatO2, oxygen saturation; WBC, white blood count

Gastrointestinal symptoms encompassed: nausea, vomiting, diarrhoea or abdominal pain; mucocutaneous involvement encompassed: rash, erythema at BCG site, conjunctivitis, hands and feet erythema or oedema, digital peeling, inflammation of the oral cavity or cervical lymphadenopathy; upper respiratory symptoms encompassed: coryza or sore throat; lower respiratory symptoms encompassed: cough, breathing effort, chest pain and swallowing difficulty; neurological involvement encompassed: meningeal signs, lethargy, seizures, headache, muscle hypotension, peripheral nerve paralysis, paresis, loss of smell or taste, photophobia, agitation or skin hyperesthesia; osteoarticular and muscle involvement encompassed: arthritis, arthralgia or muscle pain

## Table S5. Comparison between children with and without contractility abnormalities, coronary artery abnormalities and pericardial effusion in the course of the multisystem inflammatory syndrome in children

| Feature<br>Counts (%) or Median (25th-75th percentile) | | Patients with CAA at any time<br>n=36 | Patients with no CAA at any time<br>n=307 | p | Patients with contractility abnormalities at any time<br>n=155 | Patients with no contractility abnormalities at any time<br>n=219 | p | Patients with pericardial effusion at any time<br>n=47 | Patients with no pericardial effusion at any time<br>n=305 | p | Patients with Reduced LVEF<br>n=132 | Patients with Normal LVEF<br>n=239 | p |
|---|---|---|---|---|---|---|---|---|---|---|---|---|---|
| **Demographical features** | | | | | | | | | | | | | |
| Male sex | | 25 (69%) | 186 (61%) | 0.37 | 105 (69%) | 131 (60%) | 0.08 | 35 (76%) | 183 (60%) | **0.05** | 89 (69%) | 144 (61%) | 0.11 |
| Age (years) | | 5 (3-12) | 9 (5-12) | **0.03** | 10 (6-13) | 7 (4-11) | **0.00** | 8.2 (4.6-11.5) | 8.3 (4.9-11.9) | 0.86 | 10 (6.2-13.4) | 7.6 (4.3-11.2) | **0.00** |
| Weight (kg) | | 22.3 (14.8-35.5) | 30.0 (19.5-47.0) | 0.21 | 36.5 (22.0-57.0) | 24.0 (18.0-38.5) | **0.00** | 32.0 (18.5-54.0) | 28.60(19.5-45.0) | 0.28 | 36.9 (22.9-56.5) | 24.0 (18.0-39.0) | **0.00** |
| Height (cm) | | 114 (98-154) | 134 (115-155) | **0.01** | 141(122-165) | 128 (110-148) | **0.00** | 136.5 (110.5-155.5) | 132.5 (113.5-155.0) | 0.87 | 141 (121-165) | 129 (111-149) | **0.00** |
| BMI (kg/m^2) | | 16.5 (15.4-18.1) | 16.6 (14.9-20.0) | 0.93 | 17.7 (15.6-20.9) | 16.0 (14.7-18.8) | **0.00** | 17.3 (15.3-20.5) | 16.4 (14.9-19.6) | 0.11 | 17.6 (15.6-20.9) | 16.1 (14.7-18.9) | **0.00** |
| BMI groups | normal | 18 (53%) | 211 (76%) | **0.01** | 99 (73.3%) | 154 (77%) | 0.23 | 30 (70%) | 206 (75%) | 0.75 | 87 (76%) | 164 (75%) | 0.47 |
| | obese | 3 (9%) | 19 (7%) | | 13 (10%) | 10 (5%) | | 4 (9%) | 18 (7%) | | 10 (9%) | 13 (6%) | |
| | overweight | 9 (27%) | 33 (12%) | | 19 (14%) | 25 (12%) | | 7 (16%) | 36 (13%) | | 15 (13%) | 28 (13%) | |
| | underweight | 4 (12%) | 13 (5%) | | 4 (3%) | 12 (6%) | | 2 (5%) | 15 (6%) | | 3 (3%) | 13 (6%) | |
| **Comorbidities** | | | | | | | | | | | | | |
| None | | 34 (97%) | 268 (92%) | 0.34 | 138 (91%) | 193 (93%) | 0.69 | 45 (98%) | 266 (91%) | 0.23 | 118 (91%) | 210 (93%) | 0.54 |
| **Signs and symptoms** | | | | | | | | | | | | | |
| Days of fever | | 7 (7-9) | 7 (6-9) | 0.22 | 7 (6-8) | 7 (7-9) | **0.03** | 7 (6-9) | 7 (6-9) | 0.97 | 7 (6-8) | 7 (6-9) | **0.02** |
| Gastrointestinal symptoms | | 29 (83%) | 276 (91%) | 0.12 | 139 (92%) | 194 (90%) | 0.58 | 42 (89%) | 270 (90%) | 0.80 | 118 (92%) | 211 (89%) | 0.46 |
| Mucocutaneous involvement | | 33 (94%) | 294 (97%) | 0.36 | 148 (96%) | 209 (97%) | 0.78 | 45 (96%) | 290 (96%) | 0.69 | 126 (96%) | 228 (97%) | 1.00 |
| Upper respiratory symptoms | | 13 (38%) | 111 (38%) | 1.00 | 49 (32%) | 78 (38%) | 0.27 | 18 (40%) | 105 (36%) | 0.62 | 38 (30%) | 87 (39%) | 0.08 |
| Lower respiratory symptoms | | 17 (50%) | 144 (50%) | 1.00 | 82 (54%) | 90 (45%) | 0.09 | 28 (61%) | 136 (48%) | 0.11 | 72 (56%) | 99 (45%) | 0.06 |
| Neurological symptoms | | 24 (86%) | 249 (90%) | 0.51 | 124 (95%) | 169 (87%) | **0.01** | 38 (97%) | 242 (89%) | 0.15 | 107 (95%) | 187 (88%) | 0.05 |

| | | | | | | | | | | | | |
|---|---|---|---|---|---|---|---|---|---|---|---|---|
| osteoarticular and muscle involvement | 12 (36%) | 118 (41%) | 0.71 | 51 (34%) | 86 (42%) | 0.19 | 17 (38%) | 117 (40%) | 0.87 | 42 (33%) | 95 (42%) | 0.11 |
| Systemic oedema | 0 (0%) | 2 (3%) | 1.00 | 2 (6%) | 0 (0%) | 0.21 | 0 (0%) | 2 (3%) | 1.00 | 2 (6%) | 0 (0%) | 0.16 |
| Particular symptoms | | | | | | | | | | | | |
| Arthritis | 5 (14%) | 11 (4%) | **0.02** | 5 (3%) | 12 (6%) | 0.32 | 3 (7%) | 13 (4%) | 0.46 | 5 (4%) | 12 (5%) | 0.62 |
| Conjunctivitis | 22 (61%) | 242 (81%) | **0.02** | 122 (80%) | 168 (79%) | 0.90 | 28 (61%) | 243 (81%) | **0.00** | 106 (82%) | 183 (79 %) | 0.59 |
| Eyelid swelling | 1 (7%) | 34 (36%) | **0.03** | 32 (39%) | 15 (26%) | 0.15 | 9 (47%) | 31 (31%) | 0.19 | 27 (38%) | 19 (29%) | 0.36 |
| Rhinitis | 8 (23%) | 32 (11%) | 0.05 | 13 (8%) | 24 (12%) | 0.38 | 6 (13%) | 32 (11%) | 0.63 | 10 (8%) | 27 (12%) | 0.28 |
| Oral inflammation | 20 (57%) | 210 (71%) | 0.12 | 104 (69%) | 144 (69%) | 1.00 | 26 (55%) | 207 (71%) | **0.04** | 89 (70%) | 157 (69%) | 0.91 |
| Breathing effort | 9 (25%) | 70 (24%) | 0.84 | 46 (30%) | 40 (19.%) | **0.02** | 16 (35%) | 66 (22%) | 0.09 | 41 (31%) | 46 (20%) | **0.02** |
| Chest pain | 3 (9%) | 49 (17%) | 0.32 | 33 (22%) | 22 (11%) | **0.01** | 7 (16%) | 45 (16%) | 1.00 | 30 (23%) | 25 (11%) | **0.00** |
| Abdominal pain | 22 (67%) | 247 (83%) | **0.03** | 129 (85%) | 167 (80%) | 0.17 | 35 (76%) | 240 (82%) | 0.42 | 111 (87%) | 181 (79%) | 0.07 |
| Nausea | 14 (41%) | 188 (63%) | **0.02** | 91 (62%) | 130 (61%) | 1.00 | 28 (61%) | 180 (61%) | 1.00 | 80 (64%) | 141 (61%) | 0.57 |
| Diarrhoea | 23 (64%) | 181 (60%) | 0.72 | 87 (57%) | 127 (59%) | 0.75 | 20 (43%) | 184 (61%) | **0.02** | 80 (62%) | 134 (57%) | 0.38 |
| **Vital signs at admission** | | | | | | | | | | | | |
| AVPU other than A | 3 (9%) | 13 (5%) | 0.23 | 9 (7%) | 7 (3%) | 0.20 | 3 (7%) | 13 (5%) | 0.48 | 8 (7%) | 9 (4%) | 0.30 |
| Heartrate (beats/minute) | 120 (107-137) | 124 (101-140) | 0.46 | 125 (110-135) | 120 (100-140) | 0.09 | 120 (106-139) | 120 (104-140) | 0.94 | 124 (110-132) | 120 (100-140) | 0.15 |
| Prolonged CRT (>2s) | 5 (17%) | 34 (14%) | 0.59 | 22 (18%) | 21 (12%) | 0.18 | 5 (14%) | 35 (14%) | 1.00 | 18 (17%) | 25 (13%) | 0.40 |
| Systolic blood pressure (mmHg) | 102 (87-119) | 100 (89-109) | 0.32 | 92 (84-104) | 101 (92-110) | **0.00** | 98 (89-103) | 100 (89-110) | 0.62 | 91 (82-103) | 101 (92-110) | **0.00** |
| Hypotension | 3 (11%) | 42 (17%) | 0.59 | 40 (32%) | 15 (9%) | **0.00** | 5 (13%) | 41 (17%) | 0.65 | 34 (32%) | 19 (10%) | **0.00** |
| Respiratory rate (breaths/minute) | 24 (20-30) | 20 (18-26) | 0.34 | 22 (20-30) | 20 (18-25) | **0.00** | 22 (19-28) | 20 (18-26) | 0.13 | 23 (19-30) | 20 (18-25) | **0.01** |
| SatO2 (%) | 98 (97-99) | 98 (96-99) | 0.59 | 98 (96-99) | 98 (97-99) | **0.04** | 97 (96-98) | 98 (96-99) | **0.01** | 98 (96-99) | 98 (97-99) | **0.03** |
| **Laboratory test results at admission** | | | | | | | | | | | | |
| WBC (10^3/µl) | 10.8 (6.4-17.3) | 9.6 (6.6-12.8) | 0.06 | 10.4 (7.1-13.7) | 9.6 (6.2-13.2) | 0.49 | 10 (7.5-14.6) | 9.6 (6.5-13.2) | 0.50 | 10.5 (7.1-13.7) | 9.6 (6.4-13.2) | 0.43 |

| | | | | | | | | | | | | |
|---|---|---|---|---|---|---|---|---|---|---|---|---|
| Neutrophils (10^3/µl) | 8.6 (4.2-12.9) | 7.7 (4.9-10.7) | 0.38 | 8.7 (5.8-11.3) | 7.5 (4.7-10.0) | 0.05 | 7.6 (5.3-11.0) | 7.7 (4.9-10.8) | 0.66 | 8.8 (5.8-11.5) | 7.6 (4.7-10.0) | **0.03** |
| Lymphocytes (10^3/µl) | 1.3 (0.7-2.7) | 1.0 (0.7-1.6) | **0.00** | 0.8 (0.6-1.2) | 1.2 (0.7-2.0) | **0.00** | 1.0 (0.7-1.7) | 1.0 (0.7-1.7) | 0.69 | 0.9 (0.6-1.2) | 1.1 (0.7-1.9) | **0.00** |
| Hb (g/dl) | 11.2 (10.5-12.2) | 11.7 (10.7-12.7) | 0.18 | 11.9 (10.8-12.8) | 11.5 (10.7-12.6) | 0.19 | 11.9 (10.5-12.8) | 11.5 (10.7-12.6) | 0.75 | 11.9 (11.0-12.9) | 11.5 (10.6-12.5) | 0.06 |
| Hct (%) | 32.7 (30.9-35.0) | 34.0 (30.8-37.0) | 0.14 | 34.5 (30.5-37.2) | 33.3 (30.9-36.4) | 0.80 | 34.5 (30.6-36.6) | 33.8 (30.8-36.7) | 0.89 | 34.5 (30.5-37.6) | 33.3 (30.7-36.3) | 0.43 |
| PLT (10^3/µl) | 191 (151-325) | 172 (122-244) | **0.01** | 167 (122-216) | 186 (127-273) | **0.00** | 158 (114-239) | 180 (127-248) | 0.89 | 167 (122-214) | 182 (127-273) | **0.00** |
| CRP (mg/l) | 121 (55-173) | 145 (91-205) | 0.07 | 171 (102-240) | 137 (84-188) | **0.00** | 164 (86-232) | 143 (87-196) | 0.23 | 176 (103-246) | 138 (87-188) | **0.00** |
| ESR (mm/h) | 60.5 (42.0-85.0) | 46.5 (29.5-66.0) | 0.12 | 48.0 (30.5-65.5) | 46.5 (29.5-70.0) | 0.63 | 42 (24-61) | 48 (30-70) | 0.26 | 50.0 (33.0-68.0) | 46.0 (29.0-66.0) | 0.29 |
| Fibrinogen (g/l) | 5.5 (4.8-6.8) | 5.5 (4.5-6.5) | 1.00 | 5.7 (4.7-6.7) | 5.3 (4.3-6.4) | 0.16 | 5.5 (4.2-6.9) | 5.5 (4.5-6.5) | 0.57 | 5.7 (5.0-6.7) | 5.3 (4.3-6.5) | 0.17 |
| LDH (U/l) | 261 (214-350) | 281 (236-327) | 0.41 | 288 (248-340) | 266 (227-324) | 0.0967 | 257 (209-297) | 281 (236-329) | 0.10 | 293 (248-344) | 266 (229-323) | 0.67 |
| Procalcitonin (ng/ml) | 3.2 (0.9-6.8) | 2.8 (1.1-8.6) | 0.78 | 4.6 (1.7-16.0) | 2.0 (0.8-6.2) | **0.00** | 3.8 (1.3-10.6) | 2.5 (1.0-8.3) | 0.23 | 4.9 (1.7-17.6) | 2.1 (0.9-6.3) | **0.00** |
| Ferritin (ug/l) | 354 (150-771) | 341 (197-567) | 0.87 | 435.70 (235.10-760.20) | 318 (181-532) | **0.015** | 368 (237-581) | 336 (186-576) | 0.67 | 436 (234-760) | 323 (184-534) | 0.16 |
| Serum Glucose (mg/dl) | 97 (87-113) | 100 (89-117) | 0.17 | 104 (90-119) | 99 (88-116) | 0.61 | 103 (90-124) | 100 (88-116) | 0.82 | 104 (89-123) | 99 (88-115) | 0.51 |
| Albumins (g/dl) | 3.4 (3.1-3.7) | 3.3 (2.8-3.7) | 0.64 | 3.3 (3.0-3.7) | 3.4 (2.8-3.7) | 0.74 | 3.3 (2.7-3.8) | 3.4 (2.8-3.7) | 0.79 | 3.3 (3.0-3.8) | 3.4 (2.8-3.7) | 0.29 |
| Sodium (mmol/l) | 136 (132-138) | 134 (131-136) | 0.10 | 133 (130-136) | 134 (132-136) | **0.00** | 135 (131-136) | 134 (132-136) | 0.67 | 132 (130-135) | 135 (132-136) | **0.00** |
| D-dimer (Mg/l) | 1.9 (1.4-3.9) | 2.7 (1.5-4.6) | 0.11 | 2.9 (1.9-4.6) | 2.5 (1.5-4.5) | 0.212 | 2.9 (2.1-5.0) | 2.6 (1.5-4.5) | 0.21 | 2.8 (1.7-4.4) | 2.6 (1.5-4.5) | 0.28 |
| IL-6 (pg/ml) | 77.8 (53.8-197.1) | 105 (27-321) | 0.62 | 106 (29-316) | 107 (45-314) | 0.8 | 52 (27-73) | 110 (46-321) | 0.10 | 143 (73-446) | 104 (31-303) | 0.38 |
| eGFR (ml/min/1.73m^2) | 100 (83-130) | 112 (88-134) | 0.30 | 99 (78-120) | 116 (95-137) | **0.00** | 98 (80.0-140) | 110 (89-134) | 0.96 | 97 (78-120) | 116 (93-136) | **0.00** |
| BNP (pg/ml) | 138 (39-695) | 578 (143-1381) | **0.03** | 1197 (328-2814) | 211 (135-798) | **0.01** | 818 (660-2814) | 282 (135-1012) | 0.22 | 1381 (640-2814) | 193 (122-798) | 0.45 |
| NT-proBNP (pg/ml) | 387 (220-4956) | 2845 (651-7791) | **0.04** | 5053 (1183-13597) | 1589 (424-5381) | **0.00** | 3058 (368-15995) | 2128 (544-7400) | 0.58 | 5271 (786-14657) | 1862 (455-5701) | 0.22 |
| Troponin elevated | 5 (31%) | 43 (25%) | 0.56 | 35 (49%) | 18 (15%) | **0.00** | 8 (27.6%) | 42 (269%) | 0.82 | 31 (52%) | 22 (16%) | **0.00** |
| **Management** | | | | | | | | | | | | |
| PICU treatment | 3 (8%) | 24 (8%) | 1.00 | 20 (13%) | 10 (5%) | **0.00** | 7 (15%) | 22 (7%) | 0.08 | 19 (15%) | 12 (5%) | **0.00** |
| IVIG administered | 34 (97%) | 288 (94%) | 1.00 | 153 (99%) | 202 (93%) | **0.01** | 46 (98%) | 287 (957%) | 0.71 | 130 (99%) | 222 (94%) | **0.04** |

| | | | | | | | | | | | | |
|---|---|---|---|---|---|---|---|---|---|---|---|---|
| GCS administered | 26 (74%) | 219 (74%) | 1.00 | 129 (85%) | 143 (68%) | **0.00** | 34 (76%) | 216 (74%) | 0.86 | 115 (89%) | 154 (67%) | **0.00** |
| ASA administered | 32 (100%) | 276 (100%) | | 143 (100%) | 193 (100%) | | 44 (100%) | 273 (100%) | | 121 (100%) | 213 (100%) | |
| Heparin administered | 10 (36%) | 106 (41%) | 0.69 | 53 (51%) | 64 (35%) | **0.01** | 16 (46%) | 101 (40%) | 0.58 | 46 (52%) | 71 (36%) | **0.01** |

Abbreviations: AVPU, alert, verbal, pain, unresponsive; ASA, acetylsalicylic acid; BMI, body mass index; BNP, brain natriuretic peptide; CAA, coronary artery abnormality; CRP, C reactive protein; CRT, capillary refill time; eGFR, estimated glomerular filtration rate; ESR, erythrocyte sedimentation rate; GCS, glucocorticosteroids; Hb, hemoglobin; Hct, haematocrit; Il-6, interleukin 6; IQR, interquartile range; IVIG, intravenous immunoglobulins; LDH, lactate dehydrogenase; min, minutes; n, number; NT-proBNP, N-terminal prohormone for brain natriuretic peptide; PICU, paediatric intensive care unit; PLT, platelet count; s, seconds; SatO2, oxygen saturation; WBC, white blood count

Gastrointestinal symptoms encompassed: nausea, vomiting, diarrhoea or abdominal pain; mucocutaneous involvement encompassed: rash, erythema at BCG site, conjunctivitis, hands and feet erythema or oedema, digital peeling, inflammation of the oral cavity or cervical lymphadenopathy; upper respiratory symptoms encompassed: coryza or sore throat; lower respiratory symptoms encompassed: cough, breathing effort, chest pain and swallowing difficulty; neurological involvement encompassed: meningeal signs, lethargy, seizures, headache, muscle hypotension, peripheral nerve paralysis, paresis, loss of smell or taste, photophobia, agitation or skin hyperesthesia; osteoarticular and muscle involvement encompassed: arthritis, arthralgia or muscle pain
